# Insights into the evolution of the ErbB receptor family and their ligands from sequence analysis

**DOI:** 10.1186/1471-2148-6-79

**Published:** 2006-10-06

**Authors:** Richard A Stein, James V Staros

**Affiliations:** 1 Dept. of Molecular Physiology and Biophysics, Vanderbilt University, Nashville, TN 37232, USA; 2 Dept. of Biochemistry and Cell Biology, SUNY-Stony Brook, Stony Brook, NY 11794, USA, and Dept. of Biological Sciences, Vanderbilt University, Nashville, TN, 37235, USA

## Abstract

**Background:**

In the time since we presented the first molecular evolutionary study of the ErbB family of receptors and the EGF family of ligands, there has been a dramatic increase in genomic sequences available. We have utilized this greatly expanded data set in this study of the ErbB family of receptors and their ligands.

**Results:**

In our previous analysis we postulated that EGF family ligands could be characterized by the presence of a splice site in the coding region between the fourth and fifth cysteines of the EGF module and the placement of that module near the transmembrane domain. The recent identification of several new ligands for the ErbB receptors supports this characterization of an ErbB ligand; further, applying this characterization to available sequences suggests additional potential ligands for these receptors, the EGF modules from previously identified proteins: interphotoreceptor matrix proteoglycan-2, the alpha and beta subunit of meprin A, and mucins 3, 4, 12, and 17. The newly available sequences have caused some reorganizations of relationships among the ErbB ligand family, but they add support to the previous conclusion that three gene duplication events gave rise to the present family of four ErbB receptors among the tetrapods.

**Conclusion:**

This study provides strong support for the hypothesis that the presence of an easily identifiable sequence motif can distinguish EGF family ligands from other EGF-like modules and reveals several potential new EGF family ligands. It also raises interesting questions about the evolution of ErbB2 and ErbB3: Does ErbB2 in teleosts function differently from ErbB2 in tetrapods in terms of ligand binding and intramolecular tethering? When did ErbB3 lose kinase activity, and what is the functional significance of the divergence of its kinase domain among teleosts?

## Background

The ErbB family of receptors is a diverse set of Type I receptor tyrosine kinases ubiquitously distributed throughout the animal kingdom. In vertebrates there are four family members, ErbB 1/EGF receptor, ErbB2/neu/HER2, ErbB3/HER3, and ErbB4/HER4, while in invertebrates only one receptor has been identified. The vertebrate ligands are more numerous and varied than the receptors and include, epidermal growth factor, transforming growth factor α, heparin-binding epidermal growth factor, amphiregulin, betacellulin, epiregulin, epigen, neuregulin 1–4, tomoregulin/TMEFF 1–2, and neuroglycan-C. In invertebrates, one ligand has been identified in *Caenorhabditis*, lin-3, while four ligands have been identified in *Drosophila*, vein, gurken, spitz, and keren.

We previously carried out an evolutionary analysis of the ErbB receptor and ligands [1], which was based on a more limited sequence data set than is currently available. In our analysis the order of gene duplications leading to the four mammalian receptors was supported by the known functions and interactions of the receptors, while the segregation of the mammalian ligands into EGF receptor ligands and ErbB3/ErbB4 ligands mirrored the receptor segregation. In addition, sequence comparison between different species and receptors suggested regions of the receptors that might lead to specific differences in function between the four different receptors.

Recent genomic sequencing from a variety of species should allow for a substantial expansion of the previous analysis, which focused mainly on the mammalian and specifically the human receptors. The completed or partial genomic sequences from zebrafish, fugu, tetraodon, xenopus, and chicken among other species, allow for the examination of sequence variation of additional branches of the vertebrates beyond the mammalian lineage and how these different branches compare to each other. Comparison of these additional sequences confirms our previous description of the gene duplication events for the receptors, while the additional ligands generate a more populated ligand tree that yields new perspectives about receptor specificity.

## Results and discussion

### Ligands

Our earlier analysis suggested that EGF family ligands could be distinguished from non-ligand EGF motifs based on the presence of a splice site between the fourth and fifth cysteines within the six cysteine EGF-module and the placement of this module in close proximity to the transmembrane region of the potential ligand [1]. Since our last analysis, several new ligands have been identified. One of these ligands, identified from a mouse keratinocyte expressed sequence tag library, has been termed epigen [2]. The EGF-module occurs prior to a putative transmembrane region and examination of its chromosomal location indicates a splice site between the fourth and fifth cysteines. Two other ligands are very similar and have been called either tomoregulin 1 and 2 or TMEFF (transmembrane with an egf and two follistatin domains) 2 and 1 [3,4]. Both of these ligands also have the proposed splice site and location relative to a putative transmembrane region. A report suggested that the EGF-module from neuroglycan-C is a ligand for ErbB3 [5] and it has the proposed splice site and location relative to a putative transmembrane region. The chicken homologue to neuroglycan-C, CALEB, is noted in the databank to be chicken EGF (accession # CAA70459), but was first identified as a neural member of the EGF family and was shown to be associated with glial and neuronal tissues [6]. In the invertebrates, keren was identified in *Drosophila *as a close homologue to the previously identified spitz [7]. Of the newly discovered ligands, only keren, like its extensively characterized homologue spitz, does not have the proposed splice site, which likely reflects the general reduction of introns in the *Drosophila *genome.

In addition to the previously described ligands and the newly described ligands, this study has also identified additional EGF modules in previously described proteins that have the splice site between the fourth and fifth cysteines and are near putative transmembrane domains. These modules occur in mucin 3, 4, 12, and 17, meprin 1α and 1β, and interphotoreceptor matrix proteoglycan 2. Only one of these proteins, mucin 4, has been directly implicated in the activity of the ErbB receptor family. It has been shown that mucin 4 down regulates the signaling ability of ErbB2, though not as a secreted ligand, but as a membrane bound protein [8]. Whether the other candidate ligands that we have identified act as direct ErbB receptor ligands or are capable of modulating their activity remains to be determined.

These ligands and other previously identified ligands used in the evolutionary analyses are shown in Table 1. There are several interesting points about the identified ligands and the species that are represented. The putative invertebrate ligand, argos, which was thought to be an antagonist, was omitted from this analysis since it was found to act not on the receptor, but by interacting with ligand to carry out its antagonistic activity [9]. The ligand spitz was found in several invertebrate species in addition to *Drosophila *and in these species spitz had the splice site between the fourth and fifth cysteines, unlike spitz from *Drosophila*. The newly identified keren that is highly homologous to spitz was only found in *Drosophila *and *G. morsitans*, though interestingly no spitz was identified in *G. morsitans*. This does not prove that it does not exist, simply that it was not found via homology (BLAST [10]) searches. In addition, gurken, without the splice site, was found only in *Drosophila*; whereas vein was found in several additional invertebrates, with the splice site present in all species including *Drosophila*.

**Table 1:** List of Ligands

Ligand^a^	Rec^b^	Species^c^
amphiregulin (AR)	E1	c, ch, co, d, es, f, h, m, ma, o, p, r, ra, rh, rt(2), t, xt, z
betacellulin (BTC)	E1, E4	ae, c, ca, ch, co, d, es, f, h, m, p, r, ra rh, s, t, xt, z
epidermal growth factor (EGF)	E1	ae, c, ch, ct, d, dn, et, f, h, m, me, o, p, r, t, xt, z
epigen	E1	c, ch, co, d, h, m, o, r, ra, xl(2), xt, z
epiregulin (EPR)	E1, E4	c, ch, co, d, f, h, m, me(2), o, r, ra, rh, rt(2), t, xt, zf
gurken	I	da, de, di, dm, dp, dr, ds, dw, dy
heparin-binding epidermal growth factor (HB-EGF)	E1, E4	c, eg, ch, co, d, f, gm, h, m, ma, me, o, p, r, ra, rt, st, t, xt, z
interphotoreceptor matrix proteoglycan-2 (IMP2)	UN	c, ch, co, d, f, h, m, o, r, rh, t, xt, z
keren	I	da, de, dg, di, dj, dm, dp, dr, ds, dv, dw, dy, g
lin3	I	cb, ce
meprin 1α (MEP1α)	UN	ae, c, ch, d, dn, f, h, m, o, r, rh, t, xl, xt, z
meprin 1β (MEP1β)	UN	c, ch, co, d, f, h, m, o, p, r, rh, t, xl(2), xt, z
mucin 3 (MUC3)	UN	co, h, m, r, rh, rt, xt(3), z
mucin 4 (MUC4)	P	c, co, d, h, m, o, r, xt
mucin 12 (MUC12)	UN	c, co, d, h, m, ra, rh
mucin 17 (MUC17)	UN	d, et, h, m, o, r, rh
neuregulin-1α (NRG1α)	E3, E4	ch, co, d, dn, gu, h, m, ma, o, p, r, ra, rt, xl, z
neuregulin-1β (NRG1β)	E3, E4	c, ch, co, d, h, m, o, r, xl, z
neuregulin-2α (NRG2α)	E3, E4	c, ch, co, d, et, f, gu, h, m, o, r, rh, t, z
neuregulin-2β (NRG2β)	E3, E4	c, ch, co, d, et, f, gu, h, m, o, r, rh, z
neuregulin-3 (NRG3)	E4	c, ch, co, d, es, f, gu, h, m, o, p, r, t, xt, z(2)
neuregulin-4 (NRG4)	E4	c, ch, co, f, gu, h, m, me, o, p, r, rh, rt, xt
neuroglycan-C (NGC)	E3	ab, c, ch, co, d, f, h, m, me(2), o, r, rh, sh, xt, z
spitz	I	an, da, de, dg, di, dj, dm, dp, dr, ds, dw, dv, dy, hb, 1, tc, yf
tomoregulin-1 (TR1)	E4	c, ch, co, d, dn, es, et, f, h, m, o, p, r, xl, xt, z(2)
tomoregulin-2 (TR2)	E4	c, ch, co, d, f, h, m, o, p, r, rh, rt, t(2), xl, xt, z
transforming growth factor α (TGFα)	E1	aa, ae, c, ch, co, d, dn, f, h, m, ma, o, or, p, r, ra, rh, sh, t, xl(2), xt(2), z
vein	I	an, da, de, dg, di, dj, dm, dp, dr, ds, dv, dy, hb, yf
viral growth factor	V	ar, be; bp(2); cl(2), cp, ep(5), fp(2), gp(2), ls(2), mp, my, rf, rp, sa, sp(3), va(4), vc(5), yl

^a ^List of ligands used in the evolutionary analysis. In parentheses are the abbreviated names used in the text and figures.

^b ^Indication of the receptor that each ligand binds to. E1: EGF receptor; E3: ErbB3; E4: ErbB4; I: the invertebrate ligands bind to the one invertebrate receptor; P: interacts with ErbB2, but it is unknown if it is as a typical ligand; V: the receptor specificity varies and depends on the origin of the poxvirus growth factor; UN: the receptor specificity for these potential ligands is unknown.

^c ^List of species for each ligand. The number in parentheses indicates the number of copies of the ligand found in that species or in the case of the viral growth factors the number of viral strains. The abbreviations used are as follows:

aa: *Arvicanthis ansorgei*; ab: *Astatotilapia burtoni*; ae: *Loxodonta africana *(African elephant); an: *Anopheles gambiae *(African malaria mosquito); ar: aracatuba; be: BeAn58058; bp: cowpox; c: *Pan troglodytes *(chimp); ca: *Cyprinus carpio *(carp); cb: *Caenorhabditis briggsae*; ce: *Caenorhabditis elegans*; cg: *Cricetulus grises *(Chinese hamster); ch: *Gallus gallus *(chicken); cl: camelpox; co: *Bos taurus *(cow); cp: canarypox; ct: *Felis catus *(cat); d: *Canis familiaris *(dog); da: *Drosophila ananasse*; de: *Drosophila erectus*; dg: *Drosophila gritnshawi*; di: *Drosophila sitnulans*; dj: *Drosophila mojavensis*; dm: *Drosophila melanogaster*, dn: *Dasypus novemcinctus *(armadillo); dp: *Drosophila pseudoobscura*; dr: *Drosophila persimilis*; ds: *Drosophila sechellia*; dv: *Drosophila virilis*; dw: *Drosophila willistoni*; dy: *Drosophila yakuba*; ep: ectromelia; es: *Sorex araneus *(European shrew); et: *Echinops telfaira *(small Madagascar hedgehog); f: *Takifugu rubripes *(fugu); fp: fowlpox; g: *Glossina morsitans *(tsetse fly); gm: *Cercopithecus aethiops *(green monkey); gp: goatpox; gu: *Cavia porcellus *(guinea pig); h: *Homo sapiens *(human); hb: *Apis mellifera *(honey bee); 1: *Homerus americanus *(American lobster); Is: lumpy skin disease; m: *Mus musculus *(mouse); ma: *Mesocricetus auratus *(golden hamster); me: *Oryzias latipes *(medaka); mp: monkeypox; my: myxoma; o: *Monodelphis domestica *(opossum); or: *Pongo pygmaeus *(orangutan); p: *Sus scrofa *(pig); r: *Rattus novegicus *(rat); ra: *Oryctolagus cuniculus *(rabbit); rf: rabbit fibroma; rh: *Macaca mulatta *(rhesus monkey); rp: rabbitpox; rt: *Oncorhynchus mykiss *(rainbow trout); s: *Salmo salar *(salmon); sa: SPAN232; sh: *Ovis aries *(sheep); sp: sheeppox; st: *Gasterosteus aculeatus *(stickleback); t: *Tetraodon nigroviridis *(tetraodon); tc: *Tibolium castaneum*; va: variola; vc: vaccinia; xl: *Xenopus laevis*; xt: *Xenopus tropicalis*; yf: *Aedes aegypti *(yellow fever mosquito); yl: yaba-like; z: *Danio rerio *(zebrafish)

**Figure 1:**
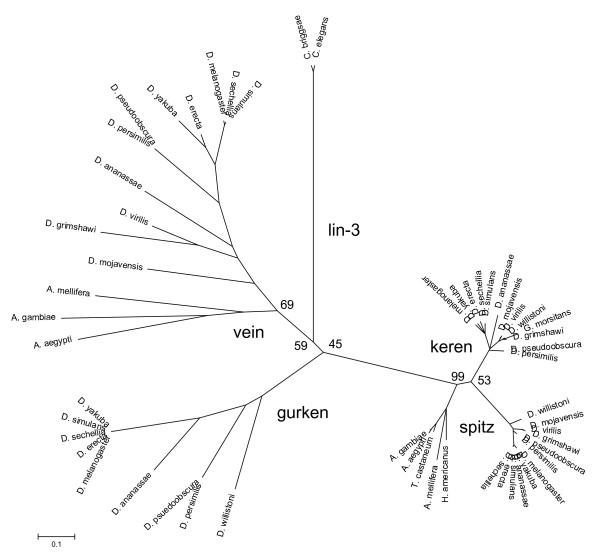
Phylogenetic relationship of the EGF modules from the invertebrate ErbB ligands. This tree was generated using neighbor joining with poisson correction of protein sequences in MEGA version 3.1 [61]. Some of the bootstrap percentages for the various branch points are shown.

ErbB family ligands are generally proteolytic cleavage products from diverse multidomain transmembrane proteins, with only the EGF module conserved across this large family of ligands. It is for this reason that the analysis was carried out only on the conserved EGF module from each of these diverse ligand precursors. A potential downside of this approach is the loss of the statistical power of longer sequences. To address this potential problem, several trees were constructed using neighbor-joining methods with several different methods for the distance calculations. Inclusion of all the ligands yielded vastly different trees for the different methods; as a result, we examined the invertebrate and vertebrate ligand phylogenies independently. The invertebrate tree (Fig. 1) exhibits several interesting features. The tree supports the hypothesis that one ligand, represented by *Caenorhabditis *lin-3, diverged into the multiple ligands found in the other invertebrates. The strong sequence similarity between non-*Drosophila *and *Drosophila *invertebrate spitz is in agreement with spitz being the predominant EGF receptor ligand in *Drosophila *growth and development [11,12]. Interestingly, the function of keren in *Drosophila *is still unclear. At the other end of the tree is the secreted ligand vein that exhibits more sequence variability between species than does spitz. Similar ligands were found in species in addition to *Drosophila*, but it remains to be seen if vein from these species is also a secreted ligand. The divergence of vein, the absence of gurken in other invertebrates, and the closely related spitz and keren suggest interesting branch points in developmental evolution of the invertebrates.

**Figure 2:**
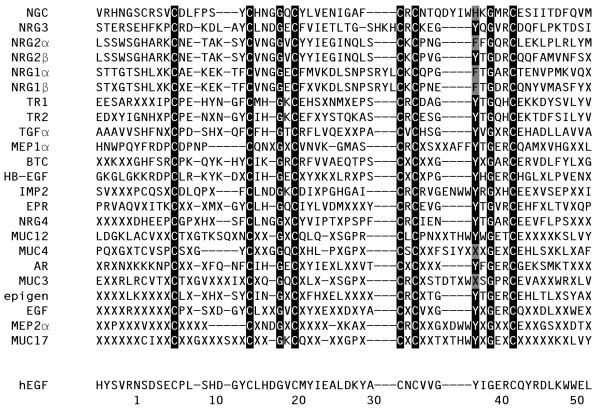
Consensus sequences for the mammalian ligands. Alignment was generated in ClustalX [60]. To minimize errors in amino acid sequence from the DNA sequences used in the analysis, a conserved residue was called conserved if it was in at least 75% of the sequences for an individual ligand. In the alignment, gaps are denoted by a dash (-) and non-conserved residues are indicated by an X. Reverse text (white text on black background) denotes residues that are at least 75% conserved among the different ligands, with grey shaded text (black text on grey background) denoting residues that are different at these conserved positions. Shown for comparison at the bottom is the sequence of human EGF and numbering for the mature ligand.

The vertebrate ligands and potential ligands in Table 1 were used to construct consensus sequences (Fig. 2). The conservation observed within each ligand for the canonical ErbB3/ErbB4 ligands is generally higher than the conservation observed within each ligand for the canonical EGF receptor ligands. How does the extent of conservation translate into function or survivability, since a higher conservation rate would suggest less tolerance for mutations? Examination of mice that have been made null for some of the ligands shows that only NRG1 is embryonic lethal with cardiac and nerve defects [13]. There are two ligands, HB-EGF and NRG2, the absence of which results in postnatal lethality [14-16], while knockouts of BTC [14], AR [17], EGF [17], EPR [18,19], TGFα [20,21], NGC [22], and the triple null AR/EGF/TGFα [17] are all non-lethal, at least under laboratory conditions. TGFα and NGC are the only ligands tested so far that are highly conserved but when absent are not lethal. In NGC null mice the defects were in synaptic transmission and the females exhibit a decrease in caring for their litters [22]; these defects could result in decreased survival outside of the laboratory environment. Mice null for TGFα do not display any deficit in fertility or lactation [20,21]. The high degree of conservation of TGFα is not due to a low number of sequences used to derive the consensus sequence or the 75% cutoff used to minimize the effect of sequencing errors, so the absence of a profound effect of a knockout of TGFα is surprising. One possibility is that TGFα mutations may have effects on viability of either the parent or offspring that are not apparent in the controlled laboratory environment.

**Figure 3:**
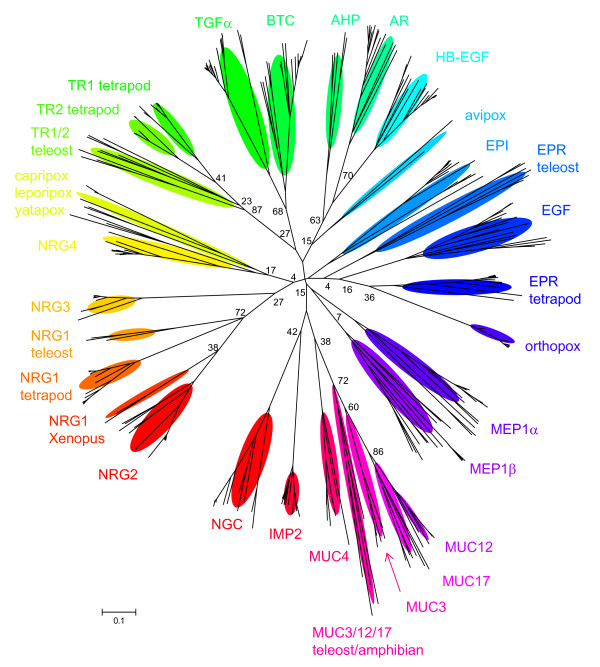
Phylogenetic relationship of the EGF modules from the vertebrate ErbB ligands. The tree shown was generated using neighbor joining with poisson correction of protein sequences in MEGA version 3.1 [61]. Each colored oval highlights the cluster of branches for a different ligand. Shown are some of the bootstrap percentages for the split between the two ligand families. Though the bootstrap percentages show low confidence in some of the branches of the tree, trees generated using different methods of distance correction exhibited similar separation of EGF receptor ligands and ErbB3/ErbB4 ligands and the positions of ligands relative to each other were comparable. Similar trees were generated using the Phylip [62] group of programs.

An unrooted tree with the labeled ligand family branches is shown in Figure 3. There are some differences in the tree depending on the method of generating the tree; however, certain features persist regardless of the method of analysis. Generally the tree segregates into EGF receptor ligands and ErbB3/ErbB4 ligands as seen previously [1], with NGC segregating with IMP2 and the mucins. The specific placement of epigen within the EGF receptor ligand branch depends on the method of generating the tree, while the other newly identified ligand, NGC, segregates with IMP2 and the mucins near the split between the EGF receptor and ErbB3/ErbB4 ligands, interesting considering the characterization of NGC as only binding to ErbB3 [5]. The two tomoregulins segregate together on what appears to be the EGF receptor portion of the tree (Fig. 3); however, an initial characterization of tomoregulin1 suggested that it was able to stimulate only ErbB4 [4]. This placement might be due to the method of analysis in constructing the tree, yet the different methods of generating the tree yielded the same placement of TR1 and TR2 near the BTC/TGFα pair. One interesting feature of the tomoregulins is a histidine prior to the sixth cysteine that is an arginine in all the other proteins that have been verified as a ligand (Fig. 2), which might alter its receptor interaction in an unknown way. Interestingly, one of the additional putative ligands identified in this study, IMP2, also has a histidine at this position, while another, MUC12, has a threonine, and three others, MEP2α, MUC4, and MUC17 are variable at this position.

There are several additional features of the tree that are worth noting. One is the placement of the viral ligands within the tree. The orthopox ligands segregate with the EGF/EPR pair, avipox segregates with AR/HB-EGF, while the leporipox, yatapox, and capripox ligands segregate with NRG4. This segregation mirrors the ligand binding properties of the shope fibroma and myxoma growth factors (leporipox) that were found to bind to ErbB3 in the presence of ErbB2, though the shope fibroma growth factor was also able to bind to ErbB1, while vaccinia growth factor (orthopox) bound to ErbB1 [23]. The variola growth factor (orthopox) was also found to only interact with ErbB1 [24]. The different positions and binding specificities of the viral ligands raise questions of viral evolution, specifically with regard to viral hosts and reservoirs and when the different viruses acquired the different ligands. Additionally, the sequence analysis and tree generation suggests that the proteins termed muc3 for rat and mouse in NCBI (AAB83956 and AAH46639, respectively, but there are multiple accession numbers for mouse) are actually muc17 as has been detected in the automated protein screens for mouse (XP_355711). In addition the teleost amphibian mucins 3, 12, and 17 segregate separately from the rest of the mucins 3, 12, and 17. The branching pattern of these three mucins is comparable to a recent analysis of mucin phylogeny using different domains from the mucins [25].

Another feature of the tree is the apparent pairing of the ligands, suggestive of gene duplication events. Within the EGF receptor ligand branches these pairs include TR1/TR2, TGFα/BTC, AR/HB-EGF, and EPR/EGF. One interesting point about these apparent gene duplications is the differential receptor specificity for binding within each pair (Table 1). With the exception of the tomoregulins, which do not appear to follow this pattern, within each pair one is more specific for the EGF receptor (TGFα, AR, and EGF), while the other has a broader receptor specificity (BTC, HB-EGF, and EPR). Although, the functional significance of this apparent cross-specificity between ligand pairs is still unclear, it is suggestive of co-evolution of the ligands and receptors and the retained interdependent function after gene duplication in this family of receptors and ligands.

**Figure 4:**
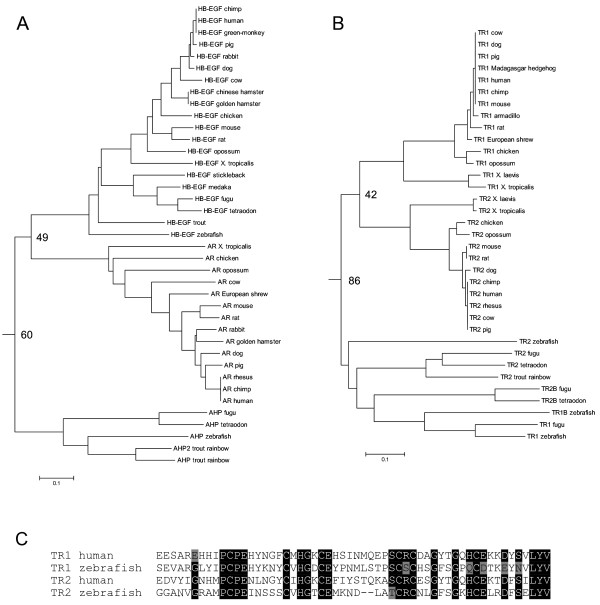
Detailed trees for the AR/HB-EGF and TR1/TR2 pairs. (A) The AR/HB-EGF pair from the tree in Fig. 3. HB-EGF exists in both teleosts and tetrapods, but there is no teleost AR, while the teleosts do have a second sequence labeled AHP, which is slightly more similar to HB-EGF than to AR. (B) The TR1/TR2 pair from the tree in Fig. 3. This tree shows an additional duplication pattern with both TR1 and TR2 forms in the teleosts segregating together. This tree is complicated by the different ligand length for TR2 in the teleosts compared to the rest of the ligands on this branch. The difference in length does suggest an alteration in the sequence requirement for TR2 in the teleosts. (C) Tomoregulin 1 and 2 sequences from human and zebrafish. These sequences are representative of the sequences from other species. TR2 is two amino acids shorter in the zebrafish than the other sequences. Reverse text (white text on black background) denotes residues that are at least 75% conserved between the four ligands, with grey shaded text (black text on grey background) denoting residues that are different at these conserved positions.

Some of the pairs that branch identically in the different trees are TGFα/BTC, AR/HB-EGF, NGC/IMP2 and TR1/TR2. While other pairs also segregate together, they do not have as high as similarity in the different trees as these pairs do. The branching patterns of the different pairs suggest different evolutionary pathways of the ligand pairs, and the different patterns might suggest different functions in the various species. The TGFα/BTC pair exhibits a simple branch with TGFα from all species examined on one side of the branch point and BTC from all species examined on the other side of the branch point, suggesting that the duplication event occurred prior to divergence of the vertebrate species examined (data not shown). This branching pattern is also seen for the NGC/IMP2 pair. The AR/HB-EGF branching exhibits a particularly interesting branching pattern (Fig. 4A). For this pair, the apparent teleost AR homologue, AHP, is actually more similar to HB-EGF than AR and branches off first. There are several possible explanations for this tree form that depend on differential sequences of gene duplications and speciation. The main point from any of the potential orders of gene duplications is that there is no direct homologue to tetrapod AR in teleosts, and conversely, there is no direct AHP homologue in tetrapods.

The TR1/TR2 pair has a different branching pattern (Fig. 4B), with both ligands in the teleost lineage segregating together and both tetrapod ligands segregating together. This pattern of branching could suggest independent gene duplications after the divergence of the two lineages or one gene duplication event that created the two ligands that then diverged with the divergence of the teleosts and tetrapods. It is noteworthy that the sequences labeled TR2 in the teleost lineage are two residues shorter than teleost and tetrapod TR1 and tetrapod TR2, which are the same length (Fig. 4C), supporting a difference in the requirement for sequence constancy between the two lineages, but it is unclear how this relates to the potential gene duplication events. In this comparison there are only sequences from teleosts and tetrapods, inclusion of sequences from additional orders might help differentiate these different possibilities. These different patterns of ligand evolution for the AR/HB-EGF and TR1/TR2 pairs argue against the indiscriminate extrapolation of function that the ligand might have in teleosts to its function in higher vertebrates, though this does not preclude a ligand from divergent lineages from having similar functions.

### Receptors

**Figure 5:**
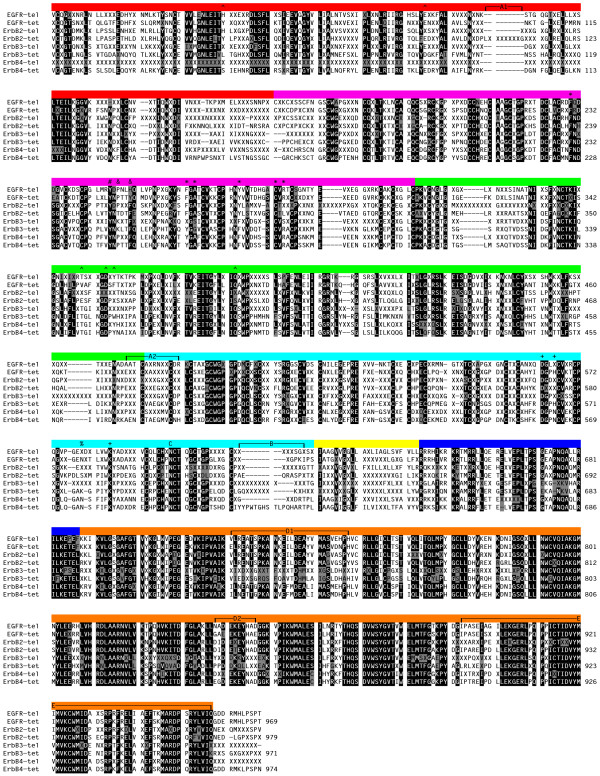
Consensus sequences for the teleost and tetrapod ErbB receptors. The alignment was generated in ClustalX [60]. To minimize errors in amino acid sequence from the DNA sequences used in the analysis, a conserved residue was called conserved if it was in 75% of the sequences. In the alignment, gaps are denoted by a dash (-) and non-conserved residues are indicated by an X. Reverse text (white text on black background) denotes residues that are at least 75% conserved between the different ligands, with grey shaded text (black text on grey background) denoting residues that are different at these conserved positions. The color bars along the top denote different subdomains within the receptor: red, subdomain I; magenta, subdomain II; green, subdomain III; cyan, subdomain IV; yellow, transmembrane; blue, intracellular juxtamembrane domain; and orange, kinase domain. The sequences start at the beginning of the second exon, and the residue numbers are for the human receptors. The regions or residues of interest are: (A) extended regions that are not well conserved in ErbB2 sequences; (B) extracellular juxtamembrane region that is alternatively spliced in ErbB4 yielding a long and short form; (C) the one glycosylation site that is conserved in the four receptors; (D) regions in the kinase domain where ErbB3 differs relative to the other three receptors, corresponding to the C-helix (D1) and the activation loop (D2); (E) the C-terminal portion of the kinase domain that has receptor-specific sequences and has been shown to be involved in mediating high affinity binding; (#) residue involved in subdomain II-subdomain II interactions in the receptor dimer and subdomain II-subdomain IV interactions in the tethered receptor monomer; (&) and (*) residues involved in subdomain II-subdomain II interactions in the receptor dimer; (+) residues involved in subdomain II-subdomain IV interactions in the tethered receptor monomer; and (^) residues that interact with ligand.

**Figure 6:**
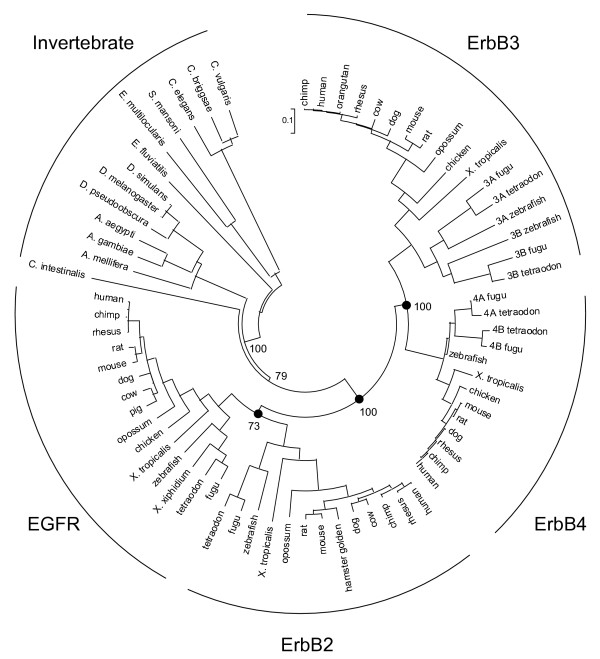
Phylogenetic relationship of the ErbB receptors. Shown is a tree generated using neighbor joining with p-distance correction of protein sequences in MEGA version 3.1 [61]. Shown are the bootstrap percentages for the split between invertebrate and vertebrate receptors. Similar trees were generated using different methods of distance correction. The invertebrate receptors lead into the vertebrate receptors separating ErbB3 and ErbB4 from EGF receptor and ErbB2. This structure suggests three gene duplication events, depicted by the filled circles, the first generating EGF receptor/ErbB2 and ErbB3/ErbB4 progenitors. Two more gene duplication events generated the four receptors seen in the vertebrates.

Unlike the ligands, no new members of the ErbB receptor family have been identified since our earlier analysis [1], only receptors from additional species. A list of the species for each of the four receptors used in the following analyses is given in Table 2. Figure 5 shows the consensus sequences for teleosts and tetrapods of the four vertebrate receptor subtypes for the extracellular domain through the kinase domain. The C-terminal regions were omitted because they are highly divergent among the different receptors, though they were included in the construction of trees for the receptors (Fig. 6). As for the ligands, several methods were used to construct unrooted trees for the receptors, but unlike the ligand trees, there is no significant difference in the trees from the different methods used, and all methods yield a tree similar to that previously constructed [1]. The additional sequences used to construct this tree support the notion that three gene duplication events generated the four receptors seen in vertebrates (Fig. 6). The first gene duplication generated ErbB1/ErbB2 and ErbB3/ErbB4 precursors. The presence of one receptor in the deuterostome invertebrate *C. intestinalis *supports the placement and the timing of the two large scale gene duplication events in the early divergence of the vertebrates [26,27]. The ErbB1/ErbB2 and ErbB3/ErbB4 precursors each underwent a second gene duplication event to generate the four receptors present in vertebrates. In addition, both ErbB3 and ErbB4 underwent an additional round of gene duplication in the teleosts, as evidenced by the two copies of each of these receptors [28]. These gene duplication events raise issues about the functional interactions of the four tetrapod receptors. It is known that the receptors undergo heterodimerization and that this heterodimerization is functionally relevant, suggesting that conservation of the ability to form functional heterodimers must have played a role in the evolution of the current receptors with their interdependent functions. ErbB3 has an inactive kinase [29,30], but it is still required for functional development [31,32]. ErbB2 has no known ligand, but it still functions as a dimerization partner [33,34]. The conservation within each of these two receptors across species supports the functional importance for the differences between receptor subtypes, but the differences within receptor subtypes across species (discussed below) raise questions as to when these functional differences might have arisen. Further investigation of the function of the receptors in various species should yield insights into the question of when these functional differences arose.

**Table 2:** List of Receptors

Receptor^a^	Species^b^
EGF receptor	an, c, cb, ce, ch, ci, co, cv, d, dm, dp, ds, ef, em, f, h, hb, m, op, p, r, rh, sm, t, xt, xx, yf, z
ErbB2	ch, co, d, f, h, m, ma, op, r, rh, t, xt, z
ErbB3	c, ch, co, d, f(2), h, m, o, op, r, rh, t(2), xt, z(2)
ErbB4	c, ch, d, f(2), h, m, r, rh, t(2), xt, z

^a^List of receptors used in the evolutionary analysis.

^b^List of species for each receptor. The number in parentheses indicates the number of copies of the receptor found in that species. The abbreviations used are as follows:

an: *Anopheles gambiae *(African malaria mosquito); c: *Pan troglodytes *(chimp); cb: *Caenorhabditis briggsae*; ce: *Caenorhabditis elegans*; ch: *Gallus gallus *(chicken); ci: *Ciona intestinalis*; co: *Bos taurus *(cow); cv: *Caenorhabditis vulgaris*; d: *Canis familiaris *(dog); dm: *Drosophila melanogaster*; dp: *Drosophila pseudoobscura*; ds: *Drosophila simulans*; ef: *Ephydatia fluviatilis*; em: *Echinococcus multilocularis*; f: *Takifugu rubripes *(fugu); h: *Homo sapiens *(human); hb: *Apis mellifera *(honey bee); m: *Mus musculus *(mouse); ma: *Mesocricetus auratus *(golden hamster); mo: *Anopheles gambiae *(mosquito); o: *Pongo pygmaeus *(orangutan); op: *Monodelphis domestica *(opossum); p: *Sus scrofa *(pig); r: *Rattus novegicus *(rat); rh: *Macaca mulatta *(rhesus monkey); sm: *Schistosoma mansoni*; t: *Tetraodon nigroviridis *(tetraodon); xt: *Xenopus tropicalis*; xx: *Xiphiphorus xiphidium*; yf: *Aedes aegypti *(yellow fever mosquito); z: *Danio rerio *(zebrafish)

The availability of two crystal structures with different ligands [35,36] aids in an initial analysis of co-evolution of ligands and receptors. This analysis is complicated by the fact that the two ligands within the dimer do not interact in an identical manner with each receptor monomer. We will focus on several residues within the receptor that interact with ligand in both structures, Tyr45, Glu90, Val350, Asp355, Phe357, and Gln384 (Fig. 5, residues labeled ^; EGF receptor numbering). A summary of the amino acids in these positions in the different receptor classes is in Table 3. In the crystal structures, Tyr45 interacts with Arg22 in TGFα or with Met21 or Ile23 in EGF depending on the monomer within the dimer (for EGF numbering see Figure 2). Arg22 and Met21 are the equivalent positions in the two ligands, but the differences in the residue from EGF (Met21 or Ile23) that interacts with the same residue in the receptor (Tyr45) highlight the malleability of the ligand-receptor interaction. The amino acid at position 90 in the receptor is mainly Glu and is in close proximity to Lys28 in EGF or Lys29 in TGFα, which are equivalent residues. While it may appear straightforward to consider the favorable ionic interaction between these oppositely charged residues, the Lys at this position is not conserved and in some instances in EGF it is a Ser. Previous mutagenic analysis of this residue has shown that while this ionic interaction between oppositely charged residues is not required for ligand binding, it does contribute to ligand affinity [37]; however, it is unclear how the specific residue present at this position within a given species affects binding. The hydrophobic Val at position 350 of the receptor interacts with Leu15 of EGF or Phe17 of TGFα, which are equivalent residues, but the hydrophobicity of the residue at receptor position 350 is not maintained across receptors. The amino acid at receptor position 355 is almost completely conserved as Asp, while it is Asn in ErbB2 from zebrafish, mouse, and golden hamster. This residue contacts Arg41 in EGF or Arg42 in TGFα, which are equivalent residues. This residue in the known ligands is also almost invariant, differing from Arg only in the tomoregulins where it is either Tyr, Gln, or His. In human EGF, mutation of Arg41 to His results in a decrease in binding; however, the observed decrease in affinity may not simply be due to a change in the interaction of this residue with Asp355 in the receptor, because this mutation also perturbs the secondary structure of human EGF [38]. Such structural effects of amino acid substitution could explain how TR1 and TR2 segregate with the canonical EGF receptor ligands (Fig. 3), but bind to ErbB4 [4]. The amino acid at receptor position 357, which is typically aromatic, interacts with Tyr13 in EGF or Phe15 in TGFα, which are equivalent residues. This residue is either Tyr or Phe across the ligands, except for TR2 in two teleosts where it is Ser. The typically polar amino acid at receptor position 384 interacts with Gln43 and Arg45 in EGF or with Glu44 in TGFα. Gln43 of EGF and Glu44 of TGFα are equivalent residues and are highly conserved within each ligand, though not necessary between ligands. These residues point to the similar binding mode of the two ligands, but it is unclear how the potential differences in binding might lead to differences in receptor homo- and heterodimerization and/or in receptor activation.

**Table 3:** Ligand/Receptor Interactions

	EGFR		ErbB2		ErbB3		ErbB4		EGF	TGFα
#^a^	tet	tel	tet	tel	tet	tel	tet	tel		

45	Tyr	His/Tyr	Tyr	His	Leu	Gln/Met	Ser	Ser	Met/Ile	Arg
90	Glu^b^	Glu^c^	Glu^d^	var	Asp^e^	Glu^f^	Glu	Glu	Lys	Lys
350	Val	Thr	Glu	var	Thr^g^	Thr^h^	Thr	Thr	Leu	Phe
355	Asp	Asp	Asp^i^	Asp^i^	Asp	Asp	Asp	Asp	Arg	Arg
357	Phe	Tyr^j^	var	var	Trp	Phe/Tyr	Tyr	Tyr^k^	Tyr	Phe
384	Gln	Gln	Ser^l^	Gln	Glu^f^	Gln	Gln	Gln	Gln/Arg	Glu

a: EGFR residue number

b: Asp in chicken, Lys in X. tropicalis

c: Asp in tetraodon

d: Gln in X. tropicalis

e: Glu in chicken

f: Asp in zebrafish

g: Ile in X. tropicalis

h: Leu in zebrafish

i: Asn in mouse, golden hamster, and zebrafish

j: His in zebrafish

k: Phe in zebrafish

l: Glu in X. tropicalis

In our previous analysis we noted the high conservation (~90% identity) between individual ErbB2 receptor sequences with two regions having less overall identity [1]. Both of these less conserved regions align with sequences in the EGF receptor that are in close proximity to bound ligand [35,36,39,40]. The addition of sequences from more diverse species does not yield new insights into the unconserved region located at the subdomain III-subdomain IV junction (Fig. 5, labeled A2), but does yield more insight into the region located in subdomain I (Fig. 5, labeled A1). This unconserved region, compared to the other three receptors, was noted as an insert in ErbB2. Interestingly, this insert does not occur in the teleosts or amphibians, suggesting that this insert occurred after the divergence of the amphibians and amniotes. It is not clear what role this insert might have in the loss of ligand binding, but it raises the question of whether the teleost or amphibian ErbB2 receptor is capable of binding ligand or whether it functions similarly to the mammalian receptor, as a dimerization partner without ligand.

The extracellular juxtamembrane region of ErbB4 also exhibits differences among species. In mammals this region exhibits alternative splicing [41] generating a long form and a short form (the long form is shown in Fig. 5, labeled B). There is a functional difference between the two isoforms, with the long form susceptible to inducible proteolytic cleavage, while the short form is insensitive to cleavage [41,42]. Interestingly, only the short form is present in teleosts. The presence of the long form of ErbB4 in the tetrapods suggests an additional function or regulation of the ErbB4 receptor in tetrapods that is not present in teleosts.

It was noted previously that only one N-linked glycosylation site (N599, EGF receptor numbering) was conserved among all of the vertebrate receptors [1]. Examination of the additional vertebrate receptor sequences currently available shows that all of these vertebrate sequences, except for the EGF receptor from *X. tropicalis*, contain this glycosylation site (Fig. 5, labeled C). It is unknown what role this glycosylation site might play in receptor maturation or function.

Since our previous analysis, the solution of crystal structures of the extracellular domains from the receptors [43-47] suggested a mechanism of ligand binding and receptor dimerization in which an intramolecular tether stabilizes the unliganded monomeric receptor and release of the tether allows a structural rearrangement permitting high affinity ligand binding and receptor dimerization [48]. There are three main extracellular regions of the ErbB receptors that are involved in either tether formation or dimerization. Two regions are in the dimerization arm of subdomain II. One region in subdomain II is involved in both interactions; it makes contact with the second region in subdomain II from another monomer to form the dimer or with subdomain IV from the same monomer to form the tether. The residues in subdomain II of one monomer that are involved in interacting with the opposing subdomain II from a second monomer are Tyr246, Pro248, and Tyr251 (Fig. 5, residues labeled # (246) and & (248, 251); EGF receptor numbering). Tyr246 is conserved in all vertebrate receptors, while the amino acid at position 248 is Pro in EGF receptor, ErbB4, and teleost ErbB2, Lys in ErbB3, and predominantly Thr in ErbB2 from tetrapods. The amino acid at position 251 is Tyr in tetrapod EGF receptor, His in teleost EGF receptor, and Phe in ErbB2, ErbB3, and ErbB4. These three residues interact with several residues in the other monomer that include, Phe230, Phe263, Ala265, Tyr275, Cys283, and Arg285 (Fig. 5, residues labeled *; EGF receptor numbering). Positions 230 and 283 are invariant, while position 263 and 275 are either Phe or Tyr; 263 is Phe in EGF receptor and ErbB2, Tyr in ErbB4 and tetrapod ErbB3, and either Phe or Tyr in teleost ErbB3, while 275 is Tyr in EGF receptor and ErbB2 and Phe in ErbB3 and ErbB4. The amino acid at position 265 is Ala in EGF receptor, ErbB2, and ErbB4, while it is Gly in tetrapod ErbB3 and Ser in teleost ErbB3. Position 285 is Arg in EGF receptor, tetrapod ErbB3, and ErbB4, Leu in ErbB2 (except for zebrafish where it is Met), and Ser in teleost ErbB3 (except for one version in tetraodon where it is Arg). This pattern of amino acids at the positions that mediate the interaction between the two monomers most likely reflects the different preferences for homo- and heterodimerization. ErbB2 and ErbB3 exhibit little to no homodimerization; differences at these sites may contribute to the inability of these receptors to homodimerize.

The tether is formed by the intramolecular interactions between subdomain II and subdomain IV. The residues involved in this interaction are Tyr246, Asp563, His566, and Lys585 (Fig. 5, residues labeled #, +; EGF receptor numbering). Tyr246 is the same residue involved in the dimer interface discussed above. The amino acids at positions 563 and 585 are invariantly Asp and Lys, respectively, while 566 is His in EGF receptor and tetrapod ErbB3, Phe in teleost ErbB2, variable in tetrapod ErbB2, His or Tyr in teleost ErbB3, and Asn in ErbB4. The high conservation of these residues suggests that tether formation occurs in all receptors, with the possible exception of tetrapod ErbB2. The potential lack of tether formation in tetrapod ErbB2 is consistent with the crystal structure obtained for ErbB2, which is in an untethered monomeric, but dimer-competent conformation. The observed conservation in teleost ErbB2 of residues involved in tether formation raises the question as to whether it has the ability to form the tether and therefore functions differently than tetrapod ErbB2. This issue was raised earlier in consideration of the insert present in the ligand binding region of tetrapod ErbB2 but not in teleost ErbB2.

Mutagenic analyses of the receptor have shown that tether formation is important in ligand affinity [43,49,50]. It has recently been shown that the extent of tethering of the monomeric receptor can be measured with an antibody (m806) that recognizes a sequence in the EGF receptor that is not accessible in either the tethered monomeric state or the dimeric state [51]. In addition, alteration of the sugar moieties affects the tethered state, with a decrease in oligosaccharide processing present in mutant or overexpressed receptors leading to an increase in the amount of untethered receptor [52]. This suggests a potential role of receptor processing in receptor signaling. Recently, it was shown that in A431 epidermoid carcinoma cells there is incomplete glycosylation at Asn579 (EGF receptor numbering) [53], a site that is conserved only in tetrapod EGF receptor (Fig. 5, residue labeled %). Mutagenesis of this consensus glycosylation site (Asn579Gln) showed that the receptor without glycosylation at this site was more untethered than wt EGF receptor and had altered ligand binding, suggesting that the tethered receptor is stabilized by the presence of the N-linked oligosaccharides at this site [54]. This might suggest that compared to the other receptors in the family, the tetrapod EGF receptors may have acquired an additional method of regulating signaling by modulating the extent of intramolecular tethering by glycosylation at Asn579.

The other regions previously highlighted fall within the kinase region of the receptors. We noted a lack of conservation in two regions within the kinase domain of the human receptors that correspond to the C-helix and activation loop (Fig. 5, labeled D1 and D2, respectively). Comparison of these regions from the additional species in this study supports the lack of conservation between receptor subtypes and points to additional receptor subtype differences in these regions. For the EGF receptor, ErbB2, and ErbB4 there is complete conservation of sequences in the C-helix (Fig. 5, labeled D1) within each receptor; while the teleost ErbB3 sequences have very little conservation and the tetrapod ErbB3 sequences have nearly complete conservation. Within this region the consensus sequences from ErbB3 vary greatly from those of the other three receptors; the other three receptor subtypes are over 50% identical. Similar to the C-helix, the region in the activation loop exhibits high conservation within each receptor subtype, except for ErbB3 from teleosts, with ErbB3 sharing very little identity with the other receptors (Fig. 5, labeled D2).

The remaining region of the kinase domain that we previously examined corresponds to the c-terminal portion of the kinase domain. What was observed was not a lack of conservation within this domain, but what appeared to be receptor subtype specific differences in particular residues in this region (Fig. 5, labeled E). The present analysis supports the identification of these residues and extends this region further into the kinase domain. The intracellular portion of the receptors that has been reported to mediate high affinity binding [55-57] corresponds to this region in the kinase domain. It was thought that this region was involved in either direct protein interactions with the other kinase domain within the dimer or that this interaction was mediated by an accessory protein.

Recently, a direct protein-protein interaction for this C-terminal region in kinase activation was found [58]. Instead of forming a symmetric interaction that leads to kinase activation an asymmetric interaction was found in which only one of the kinase domains in the dimer is thought to be active at any one time. This asymmetric dimer occurs via the C-terminal region of one kinase that interacts with the C-helix and juxtamembrane region of the other kinase leading to the activation of this kinase within the dimer. These results elegantly explain certain characteristics of the ErbB receptor family, specifically the presence of the ligand-less dimerization partner ErbB2 and the kinase inactive, but functional ErbB3. While these results support the difference in the ErbB3 sequence in the C-helix compared to the other three receptors (Fig. 5, D1), the results do not explain the high conservation of these residues in tetrapod ErbB3. If this region is not needed for kinase activation, the high conservation of residues in this region would suggest that they may have another important functional role.

## Conclusion

Examination of the ErbB receptor family and their ligands from both biochemical and evolutionary viewpoints yields insights into the functioning of the receptor and ligand families. The additional ligand sequences that have become available since our earlier analysis [1] support our characterization of an ErbB receptor ligand by the presence of a splice site in the coding region for the fourth and fifth cysteines and the placement of the EGF module near the transmembrane domain. These criteria were used to identify several potential new ErbB ligands in previously identified proteins. Except for the newly identified tomoregulins (which lack the conserved Arg before the sixth cysteine) the ligands segregate into canonical EGF receptor ligands and ErbB3/ErbB4 ligands. Except for the placement of the tomoregulins, this branching pattern is suggestive of an interesting co-evolution of the ligands and receptors.

Insight into the functioning of the ErbB receptors is gained by taking into account the evolution of the receptors. The additional receptor sequences used in this analysis support the previous conclusion that three gene duplication events led to the present set of four receptors in the tetrapods. The additional sequences also raise interesting questions about when ErbB2 lost its ligand binding capability and the role that it plays as a dimerization partner. Examination of residues involved in ligand recognition supports a general model of ligand binding, but x-ray crystal structures of ErbB3 and ErbB4 with bound ligands are needed to address whether the ErbB3/ErbB4 ligands bind similarly to their receptors and how subtle differences in ligand binding lead to differences in receptor signaling.

## Methods

Protein sequences were obtained from GenBank at the National Center for Biotechnology Information, Ensembl, TIGR, or other public databases. Sequences were identified via Blast [10] searches utilizing full length receptors or EGF modules. For the ligands, only the EGF module was used because across the ligands this is the only conserved domain. These searches yielded a variety of sequences depending on the database being searched. Where these searches yielded predicted genes, comparisons of these genes to the human sequences were carried out to verify that the predicted genes were complete. This was especially important for receptor searches, since the automated gene predications can skip exons, especially short ones. The skipped exons were then identified in the parental DNA (contig, scaffold, or higher order sequence compilation) and these were then used to construct full length DNA sequences. Where only locations in the parental DNA were found, GENSCAN [59] was used to identify exons and splice sites. If in this procedure any exons were missed, the same procedure described above was carried out to obtain full length DNA sequences. The quality of the sequences used ranged from cDNA and est sequences up to at least 7X genomic coverage. This leads to the potential that proteins used in the analysis will have a certain error rate inversely proportional to the quality of the sequencing data. All DNA sequences (see Additional file 1 for accession numbers) were converted to amino acid sequences for subsequent analyses. Consensus sequences were derived by comparing the sequences at individual positions and calling that position conserved if the percentage of the most likely amino acid occurred above the desired threshold. In defining a consensus sequence, a residue only had to be in 75% of the sequences to take into account the potential errors in the sequences. The use of the 75% cutoff balances the potential for calling a residue conserved when it really is not against calling a residue not conserved due to poor sequence quality when it is conserved. Protein alignments were carried out using ClustalX [60] with no adjustment of the default parameters. Bootstrapping (500 replicates) was carried out using MEGA (version 3.1) [61] or the Phylip group of programs (version 3.5) [62] using neighbor-joining or minimum evolution methods and several models of amino acid substitution, including poisson correction and Jones, Taylor & Thornton (JTT). Several methods of analysis were carried out to minimize any potential problems of carrying out a phylogenic analysis on the short EGF module used in these analyses, though this does not guarantee the accuracy of the obtained trees.

## Authors' contributions

RAS carried out all the data mining and sequence comparison analysis and played a central role in conceptualization of this study and in drafting the manuscript. JVS participated in conceptualization of this study and in drafting the manuscript. RAS and JVS have read and approved the final manuscript.

## Supplementary Material

Additional file 1 Ligand and receptor accession numbers. Tables listing the accession numbers and species used in the analyses. Click here for file

## References

[B1] Stein RA, Staros JV (2000). Evolutionary analysis of the ErbB receptor and ligand families. J Mol Evol.

[B2] Strachan L, Murison JG, Prestidge RL, Sleeman MA, Watson JD, Kumble KD (2001). Cloning and biological activity of epigen, a novel member of the epidermal growth factor superfamily. J Biol Chem.

[B3] Eib DW, Martens GJ (1996). A novel transmembrane protein with epidermal growth factor and follistatin domains expressed in the hypothalamo-hypophysial axis of Xenopus laevis. J Neurochem.

[B4] Uchida T, Wada K, Akamatsu T, Yonezawa M, Noguchi H, Mizoguchi A, Kasuga M, Sakamoto C (1999). A novel epidermal growth factor-like molecule containing two follistatin modules stimulates tyrosine phosphorylation of erbB-4 in MKN28 gastric cancer cells. Biochem Biophys Res Commun.

[B5] Kinugasa Y, Ishiguro H, Tokita Y, Oohira A, Ohmoto H, Higashiyama S (2004). Neuroglycan C, a novel member of the neuregulin family. Biochem Biophys Res Commun.

[B6] Schumacher S, Volkmer H, Buck F, Otto A, Tarnok A, Roth S, Rathjen FG (1997). Chicken acidic leucine-rich EGF-like domain containing brain protein (CALEB), a neural member of the EGF family of differentiation factors, is implicated in neurite formation. J Cell Biol.

[B7] Reich A, Shilo BZ (2002). Keren, a new ligand of the Drosophila epidermal growth factor receptor, undergoes two modes of cleavage. Embo J.

[B8] Ramsauer VP, Carraway CA, Salas PJ, Carraway KL (2003). Muc4/sialomucin complex, the intramembrane ErbB2 ligand, translocates ErbB2 to the apical surface in polarized epithelial cells. J Biol Chem.

[B9] Klein DE, Nappi VM, Reeves GT, Shvartsman SY, Lemmon MA (2004). Argos inhibits epidermal growth factor receptor signalling by ligand sequestration. Nature.

[B10] Altschul SF, Gish W, Miller W, Myers EW, Lipman DJ (1990). Basic local alignment search tool. J Mol Biol.

[B11] Shilo BZ (2005). Regulating the dynamics of EGF receptor signaling in space and time. Development.

[B12] Shilo BZ (2003). Signaling by the Drosophila epidermal growth factor receptor pathway during development. Exp Cell Res.

[B13] Meyer D, Birchmeier C (1995). Multiple essential functions of neuregulin in development. Nature.

[B14] Jackson LF, Qiu TH, Sunnarborg SW, Chang A, Zhang C, Patterson C, Lee DC (2003). Defective valvulogenesis in HB-EGF and TACE-null mice is associated with aberrant BMP signaling. Embo J.

[B15] Iwamoto R, Yamazaki S, Asakura M, Takashima S, Hasuwa H, Miyado K, Adachi S, Kitakaze M, Hashimoto K, Raab G, Nanba D, Higashiyama S, Hori M, Klagsbrun M, Mekada E (2003). Heparin-binding EGF-like growth factor and ErbB signaling is essential for heart function. Proc Natl Acad Sci USA.

[B16] Britto JM, Lukehurst S, Weller R, Fraser C, Qiu Y, Hertzog P, Busfield SJ (2004). Generation and characterization of neuregulin-2-deficient mice. Mol Cell Biol.

[B17] Luetteke NC, Qiu TH, Fenton SE, Troyer KL, Riedel RF, Chang A, Lee DC (1999). Targeted inactivation of the EGF and amphiregulin genes reveals distinct roles for EGF receptor ligands in mouse mammary gland development. Development.

[B18] Shirasawa S, Sugiyama S, Baba I, Inokuchi J, Sekine S, Ogino K, Kawamura Y, Dohi T, Fujimoto M, Sasazuki T (2004). Dermatitis due to epiregulin deficiency and a critical role of epiregulin in immune-related responses of keratinocyte and macrophage. Proc Natl Acad Sci USA.

[B19] Lee D, Pearsall RS, Das S, Dey SK, Godfrey VL, Threadgill DW (2004). Epiregulin is not essential for development of intestinal tumors but is required for protection from intestinal damage. Mol Cell Biol.

[B20] Luetteke NC, Qiu TH, Peiffer RL, Oliver P, Smithies O, Lee DC (1993). TGF alpha deficiency results in hair follicle and eye abnormalities in targeted and waved-1 mice. Cell.

[B21] Mann GB, Fowler KJ, Gabriel A, Nice EC, Williams RL, Dunn AR (1993). Mice with a null mutation of the TGF alpha gene have abnormal skin architecture, wavy hair, and curly whiskers and often develop corneal inflammation. Cell.

[B22] Juttner R, More MI, Das D, Babich A, Meier J, Henning M, Erdmann B, Mu Ller EC, Otto A, Grantyn R, Rathjen FG (2005). Impaired synapse function during postnatal development in the absence of CALEB, an EGF-like protein processed by neuronal activity. Neuron.

[B23] Tzahar E, Moyer JD, Waterman H, Barbacci EG, Bao J, Levkowitz G, Shelly M, Strano S, Pinkas-Kramarski R, Pierce JH, Andrews GC, Yarden Y (1998). Pathogenic poxviruses reveal viral strategies to exploit the ErbB signaling network. Embo J.

[B24] Kim M, Yang H, Kim SK, Reche PA, Tirabassi RS, Hussey RE, Chishti Y, Rheinwald JG, Morehead TJ, Zech T, Damon IK, Welsh RM, Reinherz EL (2004). Biochemical and functional analysis of smallpox growth factor (SPGF) and anti-SPGF monoclonal antibodies. J Biol Chem.

[B25] Duraisamy S, Ramasamy S, Kharbanda S, Kufe D (2006). Distinct evolution of the human carcinoma-associated transmembrane mucins, MUC1, MUC4 AND MUC16. Gene.

[B26] Meyer A, Schartl M (1999). Gene and genome duplications in vertebrates: the one-to-four (-to-eight in fish) rule and the evolution of novel gene functions. Curr Opin Cell Biol.

[B27] Panopoulou G, Poustka AJ (2005). Timing and mechanism of ancient vertebrate genome duplications – the adventure of a hypothesis. Trends Genet.

[B28] Gomez A, Volff JN, Hornung U, Schartl M, Wellbrock C (2004). Identification of a second egfr gene in Xiphophorus uncovers an expansion of the epidermal growth factor receptor family in fish. Mol Biol Evol.

[B29] Hellyer NJ, Kim HH, Greaves CH, Sierke SL, Koland JG (1995). Cloning of the rat ErbB3 cDNA and characterization of the recombinant protein. Gene.

[B30] Guy PM, Platko JV, Cantley LC, Cerione RA, Carraway KL (1994). Insect cell-expressed p180erbB3 possesses an impaired tyrosine kinase activity. Proc Natl Acad Sci USA.

[B31] Riethmacher D, Sonnenberg-Riethmacher E, Brinkmann V, Yamaai T, Lewin GR, Birchmeier C (1997). Severe neuropathies in mice with targeted mutations in the ErbB3 receptor. Nature.

[B32] Erickson SL, O'Shea KS, Ghaboosi N, Loverro L, Frantz G, Bauer M, Lu LH, Moore MW (1997). ErbB3 is required for normal cerebellar and cardiac development: a comparison with ErbB2-and heregulin-deficient mice. Development.

[B33] Klapper LN, Glathe S, Vaisman N, Hynes NE, Andrews GC, Sela M, Yarden Y (1999). The ErbB-2/HER2 oncoprotein of human carcinomas may function solely as a shared coreceptor for multiple stroma-derived growth factors. Proc Natl Acad Sci USA.

[B34] Tzahar E, Waterman H, Chen X, Levkowitz G, Karunagaran D, Lavi S, Ratzkin BJ, Yarden Y (1996). A hierarchical network of interreceptor interactions determines signal transduction by Neu differentiation factor/neuregulin and epidermal growth factor. Mol Cell Biol.

[B35] Garrett TP, McKern NM, Lou M, Elleman TC, Adams TE, Lovrecz GO, Zhu HJ, Walker F, Frenkel MJ, Hoyne PA, Jorissen RN, Nice EC, Burgess AW, Ward CW (2002). Crystal structure of a truncated epidermal growth factor receptor extracellular domain bound to transforming growth factor alpha. Cell.

[B36] Ogiso H, Ishitani R, Nureki O, Fukai S, Yamanaka M, Kim JH, Saito K, Sakamoto A, Inoue M, Shirouzu M, Yokoyama S (2002). Crystal structure of the complex of human epidermal growth factor and receptor extracellular domains. Cell.

[B37] Campion SR, Matsunami RK, Engler DA, Niyogi SK (1990). Biochemical properties of site-directed mutants of human epidermal growth factor: importance of solvent-exposed hydrophobic residues of the amino-terminal domain in receptor binding. Biochemistry.

[B38] Hommel U, Dudgeon TJ, Fallon A, Edwards RM, Campbell ID (1991). Structure-function relationships in human epidermal growth factor studied by site-directed mutagenesis and 1H NMR. Biochemistry.

[B39] Summerfield AE, Hudnall AK, Lukas TJ, Guyer CA, Staros JV (1996). Identification of residues of the epidermal growth factor receptor proximal to residue 45 of bound epidermal growth factor. J Biol Chem.

[B40] Woltjer RL, Lukas TJ, Staros JV (1992). Direct identification of residues of the epidermal growth factor receptor in close proximity to the amino terminus of bound epidermal growth factor. Proc Natl Acad Sci USA.

[B41] Elenius K, Corfas G, Paul S, Choi CJ, Rio C, Plowman GD, Klagsbrun M (1997). A novel juxtamembrane domain isoform of HER4/ErbB4. Isoform-specific tissue distribution and differential processing in response to phorbol ester. J Biol Chem.

[B42] Vecchi M, Baulida J, Carpenter G (1996). Selective cleavage of the heregulin receptor ErbB-4 by protein kinase C activation. J Biol Chem.

[B43] Ferguson KM, Berger MB, Mendrola JM, Cho HS, Leahy DJ, Lemmon MA (2003). EGF activates its receptor by removing interactions that autoinhibit ectodomain dimerization. Mol Cell.

[B44] Garrett TP, McKern MM, Lou M, Elleman TC, Adams TE, Lovrecz GO, Kofler M, Jorissen RN, Nice EC, Burgess AW, Ward CW (2003). The crystal structure of a truncated ErbB2 ectodomain reveals an active conformation, poised to interact with other ErbB receptors. Mol Cell.

[B45] Cho HS, Mason K, Ramyar KX, Stanley AM, Gabelli SB, Denney DW, Leahy DJ (2003). Structure of the extracellular region of HER2 alone and in complex with the Herceptin Fab. Nature.

[B46] Cho HS, Leahy DJ (2002). Structure of the extracellular region of HER3 reveals an interdomain tether. Science.

[B47] Bouyain S, Longo PA, Li S, Ferguson KM, Leahy DJ (2005). The extracellular region of ErbB4 adopts a tethered conformation in the absence of ligand. Proc Natl Acad Sci USA.

[B48] Burgess AW, Cho HS, Eigenbrot C, Ferguson KM, Garrett TP, Leahy DJ, Lemmon MA, Sliwkowski MX, Ward CW, Yokoyama S (2003). An open-and-shut case? Recent insights into the activation of EGF/ErbB receptors. Mol Cell.

[B49] Walker F, Orchard SG, Jorissen RN, Hall NE, Zhang HH, Hoyne PA, Adams TE, Johns TG, Ward C, Garrett TP, Zhu HJ, Nerrie M, Scott AM, Nice EC, Burgess AW (2004). CR1/CR2 interactions modulate the functions of the cell surface epidermal growth factor receptor. J Biol Chem.

[B50] Mattoon D, Klein P, Lemmon MA, Lax I, Schlessinger J (2004). The tethered configuration of the EGF receptor extracellular domain exerts only a limited control of receptor function. Proc Natl Acad Sci USA.

[B51] Johns TG, Adams TE, Cochran JR, Hall NE, Hoyne PA, Olsen MJ, Kim YS, Rothacker J, Nice EC, Walker F, Ritter G, Jungbluth AA, Old LJ, Ward CW, Burgess AW, Wittrup KD, Scott AM (2004). Identification of the epitope for the epidermal growth factor receptor-specific monoclonal antibody 806 reveals that it preferentially recognizes an untethered form of the receptor. J Biol Chem.

[B52] Johns TG, Mellman I, Cartwright GA, Ritter G, Old LJ, Burgess AW, Scott AM (2005). The antitumor monoclonal antibody 806 recognizes a high-mannose form of the EGF receptor that reaches the cell surface when cells over-express the receptor. Faseb J.

[B53] Zhen Y, Caprioli RM, Staros JV (2003). Characterization of glycosylation sites of the epidermal growth factor receptor. Biochemistry.

[B54] Whitson KB, Whitson SR, Red-Brewer ML, McCoy AJ, Vitali AA, Walker F, Johns TG, Beth AH, Staros JV (2005). Functional effects of glycosylation at Asn-579 of the epidermal growth factor receptor. Biochemistry.

[B55] Worthylake R, Wiley HS (1997). Structural aspects of the epidermal growth factor receptor required for transmodulation of erbB-2/neu. J Biol Chem.

[B56] Van der Heyden MA, Nievers M, Verkleij AJ, Boonstra J, Van Bergen en Henegouwen PM (1997). Identification of an intracellular domain of the EGF receptor required for high-affinity binding of EGF. FEBS Lett.

[B57] Schaefer G, Akita RW, Sliwkowski MX (1999). A discrete three-amino acid segment (LVI) at the C-terminal end of kinase-impaired ErbB3 is required for transactivation of ErbB2. J Biol Chem.

[B58] Zhang X, Gureasko J, Shen K, Cole PA, Kuriyan J (2006). An allosteric mechanism for activation of the kinase domain of epidermal growth factor receptor. Cell.

[B59] Burge C, Karlin S (1997). Prediction of complete gene structures in human genomic DNA. J Mol Biol.

[B60] Thompson JD, Gibson TJ, Plewniak F, Jeanmougin F, Higgins DG (1997). The CLUSTAL_X windows interface: flexible strategies for multiple sequence alignment aided by quality analysis tools. Nucleic Acids Res.

[B61] Kumar S, Tamura K, Nei M (2004). MEGA3: Integrated software for Molecular Evolutionary Genetics Analysis and sequence alignment. Brief Bioinform.

[B62] Felsenstein J (1989). Phylip – Phylogengy Inference Package (Version 3.2). Cladistics.

