# Supplementary material for: Insights into the evolution of the ErbB receptor family and their ligands from sequence analysis
Source: BMC Evol Biol. 2006 Oct 6;6:79. doi: 10.1186/1471-2148-6-79 (PMC1618406; doi:10.1186/1471-2148-6-79)
Supplement: Additional file 1 — Ligand and receptor accession numbers. Tables listing the accession numbers and species used in the analyses. [file 1471-2148-6-79-S1.pdf]

The first table is a list of species and accession numbers for ligands used in this analysis. The second table is a list of species and accession numbers for the receptors used in this analysis. The accession numbers are for sequences that can be found at TIGR or NCBI, except for some of the X. tropicalis sequences where were only the scaffolds are given, which can be found at ENSMBL. The third table is a list of species and common names used in this analysis.

AHP

D. rerio	CAAK01001584
O. mykiss	TC87850
O. mykiss	TC84154
T. nigroviridis	CAAE01014999
T. rubripes	CAAB01000047

AR

B. taurus	TC236701
C. familiaris	BQ172885
G. gallus	AY725836
H. sapiens	AADC01041658
M. auratus	Z36972
M. domestica	AAFR03038369
M. mulatta	AANU01229876+AANU01229877
M. musculus	CAAA01047051
O. cuniculus	AAGW01528153
P. troglodytes	AADA01265557
R. norvegicus	AABR03089910
S. araneus	AALT01533113
S. scrofa	TC149930
X. tropicalis	TC5852

BTC

B. taurus	TC236700
C. carpio	CF662062
C. familiaris	AAEX02019787
D. rerio	BX649525
G. gallus	AY714349
H. sapiens	AADD01047271
L. africana	AAGU0166148+AAGU01666149
M. mulatta	AANU01229895+AANU01229894
M. musculus	CAAA01110848
O. cuniculus	AAGW01203176
P. troglodytes	AADA01006256
R. norvegicus	AABR03093216+AABR03091862
S. araneus	AALT01604937
S. salar	CB515274
S. scrofa	CB477123
T. nigroviridis	CAAE01014533
T. rubripes	CAAB01005524
X. tropicalis	TC13921

EGF

C. familiaris	AB049597
D. novemcinctus	AAGV01108085
D. rerio	CAAK01020287
E. telfairi	AAIY01469128+AAIY01152384
F. catus	AB050947
G. gallus	AY588969
H. sapiens	AADD01049629
L. africana	AAGU01378537+AAGU01158457
M. domestica	AAFR03016610
M. musculus	CAAA01129155
O. latipes	BAAF02059017
P. troglodytes	AADA01122936
R. norvegicus	AABR03018963
S. scrofa	AF336151
T. nigroviridis	CAAE01014744
T. rubripes	CAAB01000836
X. tropicalis	CF345974

EPR

B. taurus	AAFC02068806
C. familiaris	AAEX02032062
G. gallus	AY582132
H. sapiens	AADC01041658
M. domestica	AAFR03038369
M. mulatta	AANU01229874
M. musculus	CAAA01047044
O. cuniculus	AAGW01469222
O. latipes	TC36781
O. latipes	BAAF02035969
O. mykiss	TC69352
O. mykiss	CA373893
P. troglodytes	AADA01135906
R. norvegicus	AABR03090450
T. guttata	CK314652
T. rubripes	CAAB01000047
T. nigroviridis	CAAE01014999
X. tropicalis	AL793276

EPIGEN

B. taurus	AAFC02032966
C. familiaris	AACN010113992
D. rerio	BX545917
G. gallus	AY885230
H. sapiens	AADC01041658
M. domestica	AAFR03038368
M. musculus	CAAA01047037
O. cuniculus	AAGW01588688
P. troglodytes	AACZ01260727
R. norvegicus	AABR03090165
X. laevis	CB198101
X. laevis	CB941979
X. tropicalis	TC12533

GURKEN

D. ananassae	AAPP01015705
D. erecta	AAPQ01007360
D. melanogaster	AABU01002759
D. persimilis	AAIZ01002387
D. psuedoobscura	AAFS01000035
D. sechellia	AAKO01001003
D. simulans	AAGH01003549
D. willistoni	AAQB01009459
D. yakuba	AAEU02000320

HB-EGF

B. taurus	AAFC02051554
C. aethiops	M93012
C. familiaris	AAEX02023203
C. griseus	AF069753
D. rerio	BX510945
G. aculeatus	CD494462
G. gallus	AF131224
H. sapiens	AADD0106389
L. africana	AAGU01083209+AAGU01506978
M. auratus	AF327896
M. domestica	AAFR03013176
M. musculus	CAAA01192109
O. cuniculus	AAGW01211180+AAGW01530530
O. latipes	BAAF02005448
O. mykiss	CX040539
P. troglodytes	AADA01233011
R. norvegicus	AABR03112836+AABR03111566
S. scrofa	Y15731
T. nigroviridis	CAAE01014573
T. rubripes	CAAB01000014
X. tropicalis	AL892919

IMP2

B. taurus	AAFC02001347
C. familiaris	AACN010294803+AACN010596043
D. rerio	BX649245
G. gallus	AB204591
H. sapiens	AADC0103738
M. domestica	AAFR03046373
M. mulatta	AANU01279653
M. musculus	CAAA01193489
P. troglodytes	AADA01326261
R. norvegicus	AABR03079183
T. nigroviridis	CAAE01014990
T. rubripes	CAAB01000660
X. tropicalis	scaffold 71

KEREN

D. ananassae	AAPP01019326
D. erecta	AAPQ01006576
D. grimshawi	AAPT01020551
D. melanogaster	AABU01002770
D. mojavensis	AAPU01011514
D. persimilis	AAIZ01003732
D. pseudoobscura	AADE01000663
D. sechellia	AAKO01000186
D. simulans	AAGH01002570
D. virilis	AANI01017344
D. willistoni	AAQB01008019
D. yakuba	AAEU02000196
G. morsitans	GMsg-6441

LIN-3

C.briggsae	CAAC01000028
C.elegans	X68070

MEP1A

C. familiaris	AAEX02010013
D. novemcinctus	AAGV01396393+AAGV01396394
D. rerio	BX510920
G. gallus	BX934901
H. sapiens	AADC01059691
L. africana	AAGU01256209
M. domestica	AAFR03002551

M. mulatta	AANU01242615
M. musculus	CAAA01049586
P. troglodytes	AACZ02071644
R. norvegicus	AABR03068055
T. nigroviridis	CAAE01015120
T. rubripes	TC5570
X. laevis	BC079789
X. tropicalis	BC089706

MEP1B

B. taurus	AAFC02022663
C. familiaris	AAEX02026422
D. rerio	AW116715
G. gallus	BU123043
H. sapiens	AADC01134237
M. domestica	AAFR03011909
M. mulatta	AANU01220983
M. musculus	CAAA01026766
P. troglodytes	AADA01224799
R. norvegicus	AAHX01093968
S. scrofa	TC149619
T. nigroviridis	CAAE01008878
T. rubripes	CAAB01002852
X. laevis	CB560023
X. laevis	CB560473
X. tropicalis	CF347053

MUC12

B. taurus	TC293918
C. familiaris	AAEX02034612
D. rerio	BX470232
H. sapiens	AADD01084229
M. mulatta	AANU01190515
M. musculus	CAAA01077340
O. cuniculus	AAGW01155921+AAGW01155920
O. mykiss	CB492403
P. troglodytes	AACZ02086294
X. tropicalis	CX971988
X. tropicalis	scaffold 749

MUC17

C. familiaris	AAEX02034611
E. telfairi	AAIY01312092
H. sapiens	AC105446
M. domestica	AAFR03049786
M. mulatta	AANU01190520
M. musculus	TC1570381
R. norvegicus	TC556079

MUC3

B. taurus	AAFC02069005
H. sapiens	AADC01156234
M. mulatta	AANU01190512
M. musculus	CAAA01077335
R. norvegicus	AAHX01071683
X. tropicalis	scaffold 749

MUC4

B. taurus	AAFC02146076
C. familiaris	AAEX02008225
H. sapiens	AADD01042047
M. domestica	AAFR03033674
M. musculus	AC139244
P. troglodytes	AACZ02045260
R. norvegicus	AABR03078600
X. tropicalis	TC3773

NGC

A. burtoni	CN469428
B. taurus	AAFC02173461
C. familiaris	AACN010329413+AACN010309183
D. rerio	CR626877
G. gallus	AF292101
H. sapiens	AADD01032477
M. domestica	AAFR03030390
M. fascicularis	AB220509
M. mulatta	AANU01238786+AANU01238787
M. musculus	CAAA01030052
O. aries	CD287687
O. latipes	BJ736164
O. latipes	TC38771
P. troglodytes	AACZ02035863+AACZ02035862
R. norvegicus	AABR03065211
T. rubripes	CAAB01001135
X. tropicalis	scaffolds 1080+7462

NRG1A

B. taurus	AAFC02078322+AAFC02018670
C. familiaris	AAEX02009416
C. porcellus	AAKN01271927+AAKN01242012
D. novemcinctus	AAGV01002390+AAGV01146429
D. rerio	BX640592
G. gallus	AC189106
H. sapiens	AADC01074894
M. auratus	U96612
M. domestica	AAFR03038185
M. musculus	CAAA01215578
O. cuniculus	AAGW01610361+AAGW01565622
O. mykiss	TC76125
R. norvegicus	AABR03100134
S. scrofa	DV227068
X. laevis	AF076618

NRG1B

B. taurus	TC256901
C. familiaris	AAEX02009416
D. rerio	BX640592
G. gallus	AC189106
H. sapiens	AADC01074894
M. domestica	AAFR03038185
M. musculus	CAAA01215578
P. troglodytes	AACZ02093700+AACZ02093701
R. norvegicus	AABR03100134
X. laevis	AF142632

NRG2A

B. taurus	AAFC02060923
C. familiaris	AAEX02023200
C. porcellus	AAKN01087419+AAKN01196894
D. rerio	CAAK01001568
E. telfairi	AAIY01402934+AAIY01422578
G. gallus	AADN01073496
H. sapiens	AADC01055468
M. domestica	AAFR03038189
M. mulatta	AANU01185871+AANU01185872
M. musculus	CAAA01055342+CAAA01219700
P. troglodytes	AACZ02064613+AACZ02064611
R. norvegicus	AABR03109537
T. nigroviridis	CAAE01010139

NRG2B

B. taurus	AAFC02060923
C. familiaris	AAEX02023200
C. porcellus	AAKN01087419+AAKN01196894
D. rerio	CAAK01001568
E. telfairi	AAIY01402934+AAIY01422578
G. gallus	AADN01073496
H. sapiens	AADC01055468
M. domestica	AAFR03038189
M. mulatta	AANU01185871+AANU01185872
M. musculus	CAAA01055342+CAAA01219700
P. troglodytes	AACZ02064613+AACZ02064611
R. norvegicus	AABR03109537
T. rubripes	CAAB01002984+CAAB01011684

NRG3

B. taurus	AAFC02063841+AAFC02065191
C. familiaris	AACN010498848+AACN010111186
C. porcellus	AAKN01377610+AAKN01190053
D. rerio	BX072570
D. rerio	CAAK01001022
G. gallus	AADN01017068+AADN01017069
H. sapiens	AADD01109260+AADD01109281
M. domestica	AAFR03024353+AAFR03024354
M. musculus	CAAA01053393+CAAA01115252
P. troglodytes	AACZ02115687+AACZ02115706
R. norvegicus	AABR03100180+AABR03102540
S. araneus	AALT01646605+AALT01238713
S. scrofa	CT954216+CT797457
T. rubripes	CAAB01000087
T. nigroviridis	CAAE01014597
X. tropicalis	CX853981

NRG4

B. taurus	AAFC02039963+AAFC02020847
G. gallus	AJ720374
guineq-S. scrofa	AAKN01573419+AAKN01205338
H. sapiens	AADC01122187
M. domestica	AAFR03043177
M. mulatta	AANU01184683+AANU01184681
M. musculus	CAAA01015131+CAAA01203237
O. latipes	TC42022
O. mykiss	CA366588
P. troglodytes	AACZ02156594+AACZ02156591
R. norvegicus	AABR03062310

S. scrofa	CJ004287
T. rubripes	CAAB01000031
X. tropicalis	

SPITZ

A. gambiae	AAAB01008981+AAAB01006792
A. mellifera	AADG05005603
A. aegypti	AAGE02017925
D. ananassae	AAPP01015718
D. erecta	AAPQ01007039
D. grimshawi	AAPT01020551
D. melanogaster	AABU01002759
D. mojavensis	AAPU01010520
D. persimilis	AAIZ01002628
D. pseudoobscura	AAFS01000472
D. sechellia	AAKO01001670
D. simulans	AAGH01009475
D. virilis	AANI01013749
D. willistoni	AAQB01006412
D. yakuba	AAEU02000279
H. americanus	CN951232
T. castaneum	AAJJ01000084+AAJJ01001850

TGFA

A. ansorgei	AY196133
B. taurus	AAFC02054578
C. familiaris	AAEX02023172+AAEX020231701
D. novemcinctus	AAGV01210382+AAGV01210385
D. rerio	AL732598
G. gallus	AY605246
H. sapiens	AADD01019202
L. africana	AAGU01126329+AAGU01126330
M. auratus	X56146
M. domestica	AAFR03004037
M. mulatta	AANU01290998
M. musculus	CAAA01102888
O. aries	L36232
P. pygmaeus	CR750285
P. troglodytes	AADA01180207
R. norvegicus	AABR03033778
S. scrofa	X71014
T. nigroviridis	CAAE01014979
T. rubripes	CAAB01002410
X. laevis	TC232011
X. laevis	BX845923
X. tropicalis	BG512749
X. tropicalis	CX938223

TR1

B. taurus	AAFC02172473
C. familiaris	AAEX02010357
D. novemcinctus	AAGV01064077
D. rerio	CAAK01000713
D. rerio	CAAK01000429
E. telfairi	AAIY01395691
G. gallus	BX933949
H. sapiens	AADD01026239
M. domestica	AAFR03009308
M. musculus	CAAA01000253+CAAA01000254
P. troglodytes	AACZ02029081
R. norvegicus	AABR03068028
S. araneus	AALT01156611
S. scrofa	AY609989
T. rubripes	CAAB01000829+CAAB01001375
X. laevis	CB561779
X. tropicalis	AL894611

TR2

B. taurus	AAFC02043942
C. familiaris	AAEX02016474
D. rerio	CAAK01000167
G. gallus	CR406492
H. sapiens	AADC01085124
M. domestica	AAFR03027317
M. mulatta	AANU01201025+AANU01201026
M. musculus	CAAA01007155
O. mykiss	TC94696
P. troglodytes	AACZ02105879+AACZ02105881
R. norvegicus	AABR03041020
S. scrofa	CV878269
T. nigroviridis	CAAE01014764
T. nigroviridis	CAAE01013871
T. rubripes	CAAB01003004
T. rubripes	CAAB01003943
X. laevis	TC227248
X. tropicalis	AL897012

VEIN

A. aegypti	AAGE02024330
A. gambiae	AAAB01008807
A. mellifera	AADG05003861
D. ananassae	AAPP01019321
D. erecta	AAPQ01006557
D. grimshawi	AAPT01020669
D. melanogaster	AABU01002770
D. mojavensis	AAPU01011565
D. virilis	AANI01017371
D. yakuba	AAEU02000150
D. persimilis	AAIZ01010184
D. pseudoobscura	AAFS01001032
D. sechellia	AAKO01000876
D. simulans	AAGH01011532
D. yakuba	AAEU02000150

VGF

aracatuba	AF503170
BeAn58058	U79140
camelpox (CMS)	AY009089
camelpox (M-96)	AF438165
canarypox	AY318871
cowpox (Brighton)	AF482758
cowpox (GRI-90)	X94355
ectromelia (Hampsted)	AJ574814
ectromelia (Ishibashi)	AJ574816
ectromelia (Moscow)	AJ574815
ectromelia (MP5)	AJ574817
ectromelia (Naval)	AJ574818
fowlpox (CV)	AF198100
fowlpox (Munich)	AJ581527
goatpox (G20-LKV)	AY077836
goatpox (Pellor)	AY077835
lumpy-skin (2490)	AF325528
lumpy-skin (Warmbaths)	AF409137
monkeypox (Zaire)	AF380138
myxoma (Lausanne)	AF170726
rabbit-fibroma (Kasza)	AF170722
rabbitpox (Utrecht)	AY484669
sheeppox (A)	AY077833
sheeppox (NISKHI)	AY077834
sheeppox (TU-V02127)	AY077832
SPAN232	AY523995
vaccinia (Ankara)	U94848
vaccinia (Copenhagen)	M35027
vaccinia (LIVP)	S61049
vaccinia (Tian)	AF095689
vaccinia (WR)	AY243312
Variola (Bangladesh)	L22579
Variola (Congo)	U18337
Variola (Garcia)	U18338
Variola (Somalia)	U18340
yaba-like	AJ293568

**EGFR-invertebrate**	
A. aegypti	AAGE02006743
A. gambiae	AAAB01008944
A. mellifera	AADG05002302+AADG05002303
C. briggsae	CAAC01000006
C. elegans	X577167
C. intestinalis	AABS01000134
C. vulgaris	D63427
D. melanogaster	AABU01002765
D. pseudoobscura	AADE01000005
D. simulans	AAGH01006527+AAGH01006528
E. fluviatilis	AB006570
E. multilocularis	AJ515524
S. mansoni	M86396
**EGFR/ErbB1**	
B. taurus	AAFC02050811+AAFC02154291+AAFC02050796+AAFC02050802
C. familiaris	AAEX02028430+AAEX02028431
D. rerio	AY154658
G. gallus	AADN01011072
H. sapiens	AADB02010507
M. mulatta	AANU01110402+AANU01110403+AANU01110404+AANU01110408
M. domestica	AAFR03021556+AAFR03021557
M. musculus	AL645532
P. troglodytes	AC146013
R. norvegicus	AABR03089981+AABR03072848+AABR03090328
S. scrofa	TC131578
T. rubripes	CAAB01002539
T. nigroviridis	CAAE01014981
X. xiphidium	AY230135
X. tropicalis	scaffold 536
**ErbB2**	
B. taurus	AAFC02026684
C. familiaris	AAEX02035020
D. rerio	CR457459
H. sapiens	AADB02018391
M. auratus	D16295
M. mulatta	AANU01103769+AANU01103770+AANU01103771
M. musculus	AL591390
M. domestica	AAFR03029920
P. troglodytes	AACZ02169303-AACZ02169306
R. norvegicus	AABR03074017
T. rubripes	CAAB01002501
T. nigroviridis	CAAE01015106
X. tropicalis	scaffold 610
**ErbB3**	
B. taurus	AAFC02056853+AAFC02105097+AAFC02056859
C. familiaris	AAEX02029679
D. rerio	AL591365
D. rerio	CR847513
G. gallus	DQ358720
H. sapiens	AC034102
M. musculus	AC117232
M. domestica	AAFR03064754
M. mulatta	AANU01281041+AANU01281042
P. pygmaeus	CR858836
P. troglodytes	AADA01180192+AADA01094662
R. norvegicus	AABR03055433
T. rubripes	CAAB01002894
T. nigroviridis	CAAE01014979
T. rubripes	CAAB01000006
T. nigroviridis	CAAE01014991
X. tropicalis	scaffold 101
**ErbB4**	
C. familiaris	AAEX02010509-AAEX02010513
D. rerio	BX927062
G. gallus	AADN01050869+AADN01050870+AADN01050872+AADN01050873+AADN01050877+AADN01050878
H. sapiens	AADB02002768+AADB02002770+AADB02002771+AADB02002773+AADB02002774+AADB02002775+AADB02002776+AADB02002779
M. musculus	CAAA01004171+CAAA01004143+CAAA01120874+CAAA01176745+CAAA01019922+CAAA01109080+CAAA01019924+CAAA01162408+CAAA01019928+CAAA01019932+CAAA01109085
M. mulatta	AANU01248267+AANU01248273+AANU01248278+AANU01248278+AANU01248280+AANU01248281+AANU01248282+AANU01248286+AANU01248287+AANU01248288
P. troglodytes	AADA01180879+AADA01227103+AADA01010492+AADA01216571+AADA01051359+AADA01051360+AADA01051362+AADA01081033+AADA01081035+AADA01081037+AADA01259992+AADA01022232+AADA01022231+AADA01022229+AADA01300979
R. norvegicus	AAHX01060605+AAHX01060609+AAHX01060611+AAHX01060612+AAHX01060613+AAHX01060614+AAHX01060617+AAHX01060620+AAHX01060636
T. nigroviridis	CAAE01014553
T. rubripes	CAAB01002275
T. rubripes	CAAB01001137+CCAB01002884
T. nigroviridis	CAAE01015004
X. tropicalis	scaffold 509

Species Used

A. aegypti	Aedes aegypti	yellow fever mosquito
A. ansorgei	Arvicanthis ansorgei	rodent
A. burtoni	Astatotilapia burtoni	chiclid
A. gambiae	Anopheles gambiae	African malaria mosquito
A. mellifera	Apis mellifera	honey bee
B. mori	Bombyx mori	silkworm
B. taurus	Bos taurus	cow
C. aethiops	Chlorocebus aethiops	African green monkey
C. briggsae	Caenorhabditis briggsae	nematode
C. carpio	Cyprinus carpio	carp
C. elegans	Caenorhabditis elegans	nematode
C. familiaris	Canis familiaris	dog
C. griseus	Cricetulus griseus	Chinese hamster
C. intestinalis	Ciona intestinalis	ascidian
C. porcellus	Cavia porcellus	guinea pig
C. vulgaris	Caenorhabditis vulgaris	nematode
D. ananassae	Drosophila ananassae	fruit fly
D. erecta	Drosophila erecta	fruit fly
D. grimshawi	Drosophila grimshawi	fruit fly
D. melanogaster	Drosophila melanogaster	fruit fly
D. mojavensis	Drosophila mojavensis	fruit fly
D. novemcinctus	Dasypus novemcinctus	nine-banded armadillo
D. persimilis	Drosophila persimilis	fruit fly
D. pseudoobscura	Drosophila pseudoobscura	fruit fly
D. rerio	Danio rerio	zebrafish
D. sechellia	Drosophila sechellia	fruit fly
D. simulans	Drosophila simulans	fruit fly
D. simulans	Drosophila simulans	fruit fly
D. virilis	Drosophila virilis	fruit fly
D. willistoni	Drosophila willistoni	fruit fly
D. yakuba	Drosophila yakuba	fruit fly
E. fluviatilis	Ephydatia fluviatilis	sponge
E. multilocularis	Echinococcus multilocularis	tapeworm
E. telfairi	Echinops telfairi	small Madagascar hedgehog
F. catus	Felis catus	cat
G. aculeatus	Gasterosteus aculeatus	three spined stickleback
G. gallus	Gallus gallus	chicken
H. americanus	Homarus americanus	American lobster
H. coagulata	Homalodisca coagulata	Glassy-winged Sharpshooter
H. erato	Heliconius erato	crimson-patched longwing
H. sapiens	Homo sapiens	human
L. africana	Loxodonta africana	African elephant
L. rubellus	Lumbricus rubellus	humus earthworm
M. auratus	Mesocricetus auratus	golden hamster
M. domestica	Monodelphis domestica	gray short-tailed opossum
M. fascicularis	Macaca fascicularis	crab-eating macaque
M. mulatta	Macaca mulatta	rhesus monkey
M. musculus	Mus musculus	mouse
O. aries	Ovis aries	sheep
O. cuniculus	Oryctolagus cuniculus	rabbit
O. latipes	Oryzias latipes	Japanese medaka
O. mykiss	Oncorhynchus mykiss	rainbow trout
P. pygmaeus	Pongo pygmaeus	orangutan
P. troglodytes	Pan troglodytes	chimpanzee
R. norvegicus	Rattus norvegicus	Norway rat
S. araneus	Sorex araneus	European shrew
S. mansoni	Schistosoma mansoni	blood fluke
S. salar	Salmo salar	Atlantic salmon
S. scrofa	Sus scrofa	pig
T. castaneum	Tribolium castaneum	red flour beetle
T. guttata	Taeniopygia guttata	zebra finch
T. nigroviridis	Tetraodon nigroviridis	pufferfish
T. rubripes	Takifugu rubripes	fugu
X. laevis	Xenopus laevis	African clawed frog
X. tropicalis	Xenopus tropicalis	western clawed frog
X. xiphidium	Xiphophorus xiphidium	swordtail platyfish
